# Effect of treatment with *Lactococcus lactis* NZ9000 on intestinal microbiota and mucosal immune responses against *Clostridium perfringens* in broiler chickens

**DOI:** 10.3389/fmicb.2023.1257819

**Published:** 2023-12-18

**Authors:** Nitish Boodhoo, Bahram Shojadoost, Mohammadali Alizadeh, Jake Astill, Shahriar Behboudi, Shayan Sharif

**Affiliations:** ^1^Department of Pathobiology, Ontario Veterinary College, University of Guelph, Guelph, ON, Canada; ^2^Bristol Veterinary School, University of Bristol, Langford, Bristol, United Kingdom

**Keywords:** intestinal immunity, *Clostridium Perfringens*, *Lactococcus Lactis*, mucosal response, chickens, T cells, macrophages

## Abstract

Alterations in intestinal microbiota can modulate the developing avian intestinal immune system and, subsequently, may impact on resistance to enteric pathogens. The aim was to demonstrate that early life exposure to *Lactococcus lactis*, could affect either susceptibility or resistance of broilers to necrotic enteritis (NE). *L. lactis* NZ9000 (*rL. lactis*) pre-treatment at 1, 7, 14 and 21 days of age (DOA) led to a significant decrease in NE lesion scores in *Clostridium perfringens* infected chickens. *C. perfringens* Infection was associated with spatial and temporal decreases in mononuclear phagocytes and CD4+ αβ T cells. However, *rL. Lactis* pre-treatment and subsequent *C. perfringens* infection led to a significant increase in mononuclear phagocytes, CD8α + γδ T, αβ T cells (CD4+ and CD8α+) and B cells (IgM+, IgA+ and IgY+), as well as IL-12p40, IFN-γ and CD40. Differential expression of interleukin (IL)-6, IL-8, IL-10, IL-13, IL-18, IL-22, and transforming growth factor (TGF)-β were observed in *L. lactis* treated chickens when compared to *C. perfringens* infected chickens. Microbiota analysis in *C. perfringens* infected chickens demonstrated an increase in abundance of Bacillota, Bacteroidota, Pseudomonadota and Actinomycetota. These findings suggests that modulation of the chicken intestinal immune system by *L. lactis* confers partial protection 30 against NE.

## 1 Introduction

Interactions between the microbiota and intestinal cells play a significant role in shaping the intestinal immune system (Rescigno, 2011). Infections with various enteric pathogens during early life can disrupt the development of normal microbial communities, hence may impact the formation of immune responses (Mowat, 2003; Rescigno, 2011). In broiler chickens, *Eimeria* mediated enteropathy, facilitates secondary bacterial infections (Park et al., 2008b; Macdonald et al., 2017). Specifically, *Clostridium perfringens* expressing an array of toxins (α-toxin, necrotic enteritis B-like toxin; NetB and TpeL) exacerbates the primary inflammatory process thereby leading to disruption of intestinal epithelial cells tight junctions (Baba et al., 1997; van Immerseel et al., 2004; Wise and Siragusa, 2005; Uzal et al., 2014; Alizadeh et al., 2021). The resulting effect is loss of intestinal epithelial barrier integrity, and progression of necrotic enteritis (NE) ulcerative lesions (Parish, 1961; Fukata et al., 1988). Presence of *C. perfringens* cells in combination with various cofactors are usually required to promote overgrowth of *C. perfringens* in the intestinal tract (Keyburn et al., 2008; Shojadoost et al., 2012; Wang et al., 2019). Modulation of the intestinal mucosa is a determinant of virulent *C. perfringens* pathogenicity (Shojadoost et al., 2012; Wang et al., 2019).

While there is no clear consensus in the manner in which *C. perfringens* mediates enteropathy, some contributors to its pathogenesis have already been defined (Keyburn et al., 2008; Park et al., 2008b; Stanley et al., 2012; Huang et al., 2018; Lee et al., 2018). Sensing of *C. perfringens* pathogen-associated molecular patterns (PAMPs) by Toll-like receptor (TLR)2, TLR4 and TLR9 is thought to mediate the proceeding intestinal inflammatory processes (Lu et al., 2009; Shojadoost et al., 2012; Kamada et al., 2013; Oh et al., 2019). The subsequent inflammatory processes, based on the increased expression of interleukin (IL)-10, IL-13, and IL-17, have guided our early understanding of *C. perfringens* immune modulatory activity (Park et al., 2008a,b; Lu et al., 2009). Therefore, expression of these cytokines can be used as markers to define any gut microbes that could contribute to or antagonize *C. perfringens* cellular proliferation and induction of NE.

Beneficial bacteria, including lactic acid producing bacteria (LAB), have been used as probiotics for control of enteric pathogens in chickens, including NE (Shojadoost et al., 2022). Among LAB, *Lactococcus lactis* strains are of note. In humans, extensive usage of *L. lactis* expressing nisin as a live probiotic ingredient in yogurt preparations has garnered the designation of generally recognized as safe (GRAS) (Bermúdez-Humarán et al., 2011). Moreover, in chickens, nisin knockout *L. lactis* strains have been used as a recombinant oral vaccine vector against various respiratory pathogens (Song et al., 2017; Lahiri et al., 2019). Importantly, there is no evidence to suggest that *L. lactis cremoris* strain (NZ9000), which is increasingly used for oral inoculation and expression of recombinant proteins, could contribute to enteropathy in chickens (Cao et al., 2013; Song et al., 2017).

Since lactic acid producing bacteria can modulate the development and effector function of the intestinal immune system through a variety of mechanisms, including pattern recognition receptors (PRR) (Mowat and Agace, 2014), the present study aimed to evaluate the effects of oral administration of *L. lactis* on the chicken gut associated lymphoid tissue (GALT). Further, the safety and efficacy of *L. lactis* was assessed in chickens infected with a virulent *C. perfringens* strain (CP1 isolate).

## 2 Materials and method

### 2.1 Animals and ethics statement

One-day-old mixed sex Cobb broilers (*n* = 120) purchased from a local producer (Curtis chicks, a division of Maple Lodge hatcheries, Guelph, Ca) were group housed throughout the experiment in specific-pathogen-free filtered air positive pressure rooms on floor pens with wood shaving. All chickens had *ad libitum* access to water and commercial feed. All animal experiments were approved (028–10,783 – ISOL and AUP 4328) by the Animal Care Committee of the University of Guelph and adhered to the guidelines for the use of animals. Experiments and analyses were performed according to the ARRIVE guidelines.

### 2.2 Bacterial strains

(i) *Lactococcus lactis subsp. cremoris* strain (NZ9000 strain from MoBiTec GmbH, Germany), a derivate of *L. lactis* subsp. cremoris MG1363 with regulatory genes (nisR, nisK) integrated into the pepN gene of MG1363 were cultured (M17 broth; Gibco, Ca) and maintained in anaerobic atmosphere (30°C and no shaking using a gas pack).
(ii) Recombinant *L. lactis* (r*L. lactis*): *L. lactis* were cultured for 2 days in G/L-SGM17B medium (M17-Broth with 0.5 M sucrose, 2.5% glycine and 0.5% glucose), washed and prepared for electroporation. *L. lactis* NZ9000 cells were transformed with the pNZ8124 plasmid (1 μg/mL; MoBiTec. NICE® expression system for *Lactococcus lactis* handbook. 2015^1^) by electroporation (0.2 cm Gene Pulser Electroporation Cuvettes; Bio-Rad, On, Canada) as recommended by the manufacturer; 2000 V, 25 μF and 200 Ω with a resulting pulse of 5 msec. Post electroporation, cells were incubated on ice for 5 min and cultured in G/L-M17B (G/L-SGM17B supplemented with 20 mM MgCl_2_ and 2 mM CaCl_2_) for 2 h at 30°C. Transformed cells were subsequently streaked on M17 agar supplemented with 3 μg/mL of chloramphenicol and colonies allowed to grow for 48 h. Transformants were confirmed by plasmid DNA extraction and gel electrophoresis. *rL. lactis* were utilized for subsequent experiments to facilitate bacterial enumeration. r*L. lactis* starter and expansion cultures were maintained (30°C and no shaking) in M17 broth supplemented with 3 μg/mL of chloramphenicol. The cells were recovered by centrifugation (8,000 × g for 15 min at 4°C) and resuspended in 1 mL of PBS. The r*L. lactis* was prepared fresh on the day of inoculation.
(iii) The netB positive *C. perfringens*, CP1 isolate, strain used in this study had been generously provided by Dr. John Prescott (University of Guelph, On, Canada) (Chalmers et al., 2008). Single colonies as selected on blood agar were used to establish stock cultures. Overnight starter cultures (37°C in aerobic condition for 15 h) of the pathogenic *C. perfringens*, CP1 isolate, strain in Cooked Meat Medium (ThermoFisher Scientific, Canada) were expanded in 3% fluid thioglycollate medium (FTG; Millipore-Sigma, Canada) and incubated at 37°C in anaerobic condition for a further 15 h.

### 2.3 Experimental design and sampling

(i) *rL. lactis* treatment: One hundred and twenty-one-day-old chicks were randomly allocated into individual groups and 91-day old chicks were subsequently inoculated by oral gavage with 500 μL of the *rL. lactis* (1.0 × 10^8^ CFU) in PBS (*n* = 60) or PBS alone (*n* = 60). Boiler chicks were again inoculated by oral gavage on day 8, 15 and day 21 of age with 500 μL of the *rL. lactis* (1.0 × 10^8^ CFU) in PBS or PBS alone. The dosage of *L. lactis* utilized in this study were optimized in a pilot study which assessed the safety and dosage and frequency of application. No effects were observed at 1.0 × 10^7^ and no additive effects were observed at 10 × 10^9^ CFU against NE lesions.
(ii) Production of NE: One week before challenge, the starter diet was replaced with a wheat-based, high protein diet containing 15% fish meal (30% crude protein). In brief, on the day of challenge, the optical density of propagated *C. perfringens* cultures was measured by spectrophotometer at 600 nm and inoculum were adjusted to 3.0 × 10^8^ CFU and stored on ice. Sixty chickens that were pre-treated with the *rL. lactis* (1.0 × 10^8^ CFU) in PBS (*n* = 30) or PBS alone (*n* = 30) were infected by oral gavage twice daily for 3 days (21 to 23 days of age: DOA). At 24 DOA, all chickens were euthanized, and lesion scoring, based on the criteria listed in Table 1, was performed as previously described (Shojadoost et al., 2012).
(iii) Sampling: Five to six broiler chicks were euthanized on a weekly basis from both *rL. lactis* and PBS pre-treated chickens for 4 weeks and intestinal tissue samples were collected from the duodenum, jejunum, ileum and cecal tonsils and stored either in ice cold PBS containing penicillin (10 U/mL), and streptomycin (10 μg/mL) for further processing or in RNAlater (Millipore-Sigma, Canada) and subsequently frozen at –80°C. Intestinal contents from the respective intestinal segments were also collected on a weekly basis and frozen at –80°C.

**Table 1:** Assessment criteria for necrotic lesion scoring in *C. perfringens* infected chickens.

Score	Lesion	Number of lesions
0	No gross lesions	-
1	Thin or friable walls, or diffuse superficial but removable fibrin	-
2	Focal necrosis or ulceration	1 to 5 foci
3	Focal necrosis or ulceration	6 to 15 foci
4	Focal necrosis or ulceration	16 or more foci
5	Patches of necrosis or ulceration 2 to 3 cm long	Variable
6	Diffuse necrosis typical of field cases	Variable, but extensive

### 2.4 Enumeration of *Lactococcus lactis*

One-hundred milligrams of duodenum, jejunum and ileum intestinal contents, as collected on a weekly basis (24 h post *rL. lactis* inoculation) from five to six chickens, were dilute in 0.5 mL of ice-cold PBS. Mixtures were thoroughly vortexed and subsequently centrifuged (400 × g for 5 min). Supernatants were collected and tenfold serial dilution were performed in PBS. One hundred microliters of the dilution series were plated in triplicates on M17 agar supplemented with 5 μg/mL of chloramphenicol (Millipore-Sigma, Canada). Colonies were allowed to grow for 24 h in an anaerobic atmosphere (30°C and no shaking using a gas pack). The next day, colonies were counted to estimate recovery and persistence of *rL. lactis*.

### 2.5 Intestinal tissue mononuclear cell preparation

Five-centimeter segments of the medial duodenum, jejunum, ileum and whole cecal tonsils were harvested from chickens and stored on ice in PBS containing penicillin (10 U/mL), and streptomycin (10 μg/mL). Each tissue was cut into 0.5 cm^2^ segments and washed vigorously three times with PBS containing penicillin (10 U/mL), and streptomycin (10 μg/mL). Tissue samples were subsequently digested (collagenase type 1; 80 U/mL at 37°C for 20 min; Millipore-Sigma, Canada) in PBS containing penicillin (10 U/mL), and streptomycin (10 μg/mL). Whole tissue digests were applied onto 40-μm BD cell strainers (BD Biosciences, Canada), and crushed through using the rubber end of a 10 mL syringe plunger (Boodhoo et al., 2016). Duodenum, jejunum, ileum and cecal tonsils cell suspension were prepared by layering (2:1) onto histopaque 1,070 (Millipore-Sigma, Canada) density-gradient centrifugation and centrifuged at 2100 rpm (600 × G) for 20 min to allow the separation of mononuclear cells. Aspirated buffy coats were washed at 1500 rpm (400 × G) for 5 min in RPMI 1640 with penicillin (10 U/mL), and streptomycin (10 μg/mL). Mononuclear cells were suspended in complete RPMI cell culture medium; RPMI 1640 medium containing 10% fetal bovine serum (Millipore-Sigma, Canada), penicillin (10 U/mL), and streptomycin (10 μg/mL). Cell number and viability were calculated using a hemocytometer, and trypan blue exclusion method. Mononuclear cells were suspended in complete RPMI cell culture medium at a density of 5 × 10^6^ cells/ml and kept on ice.

### 2.6 Flow cytometry

Following a wash in FACS staining buffer (PBS with 0.2% BSA), duodenum, jejunum, ileum and cecal tonsils mononuclear cells, 5.0 × 10^5^ cells per well in triplicates, were counter stained in specific panels as APC (mouse anti-Kul01-FITC and mouse anti-major histocompatibility complex (MHC) II-PE), T cell (mouse anti-CD3ζ-PB, mouse anti-CD4-PE-CY7, mouse anti-CD8α-FITC and mouse anti-γδTCR-PE) and B cell (mouse anti-Bu1-PB, mouse anti-IgA-PE, mouse anti-IgY-FITC, mouse anti-IgM-APC) for 15 min at 4°C in FACS staining buffer. Cells were washed (400 × g for 5 min) with FACS staining buffer and incubated for a further 10 min at 4°C with 7-AAD-PE (BDTM Pharmigen, Canada). Mononuclear cells were washed and stored in 2% paraformaldehyde (PFA) in the fridge (4°C). 5.0 × 10^5^ cells were acquired on a FACS BD Canto II flow cytometer and data were processed by FlowJo V10 software. The full list of antibodies utilized is available in Table 2.

**Table 2:** Source of avian specific antibodies utilized in this study.

Antibody name	Fluorophore	Source	Catalog number	Quantity utilized
mouse anti-Kul01	FITC	Southern Biotech, Canada	8,420–02	0.5 μL
mouse anti-MHCII	PE	Southern Biotech, Canada	8,350–09	0.5 μL
mouse anti-CD3ζ	PB	Southern Biotech, Canada	8,200–26	0.5 μL
mouse anti-CD4	PE-CY7	Southern Biotech, Canada	8,210–17	0.5 μL
mouse anti-CD8α	FITC	Southern Biotech, Canada	8,220–02	0.5 μL
mouse anti-γδTCR	PE	Southern Biotech, Canada	8,230–09	0.5 μL
mouse anti-Bu1	PB	Southern Biotech, Canada	8,395–26	0.5 μL
mouse anti-IgA	PE	Southern Biotech, Canada	8,330–09	0.5 μL
mouse anti-IgY	FITC	Southern Biotech, Canada	8,320–02	0.5 μL
mouse anti-IgM	APC	Southern Biotech, Canada	8,310–31	0.5 μL
7-AAD	Far red	BDTM Pharmigen	559,925	0.5 μL

### 2.7 Quantitative real time-PCR (qRT-PCR)

(i) RNA extraction and cDNA synthesis: Total RNA was extracted using Trizol (Trizol®, Life Technologies, Inc.) from duodenum, jejunum and ileum tissue samples and preserved using RNA later. Tissue samples (50–100 mg) were homogenized in a tube containing glass beads using Elite Bead Ruptor (Omni International, Kennesaw GA, USA) with 1 mL of Trizol and RNA was extracted according to the manufacturer’s instructions as described previously (Boodhoo et al., 2022a). RNA quantity and quality were determined using the NanoDrop® ND-1000 spectrophotometer (NanoDrop Technologies, Wilmington, DE) after DNAse treatment. Synthesis of complementary DNAs (cDNA) was carried out by reverse transcription of 1 μg of total RNA using Oligo (dT) 12–18 primers and the Super-ScriptTM First-Strand Synthesis System (ThermoFisher Scientific, Canada.) according to the manufacturer’s instructions. Template cDNA were diluted 1:10 in milliQ water and stored in -20oC until required.
(ii) SYBR green qRT-PCR: qRT-PCR was run in 384-well plates with 5 μL of cDNA (1:10 dilution), 0.25 μM of forward and reverse primers, and 10 μL of SYBR Green (Roche Diagnostic, Laval, QC, Canada) and a balance of water to 20 μL total reaction volume per well. Each reaction involved a pre-incubation at 95°C for 5 min, followed by 40 cycles of 95°C for 20 s, 55°C–64°C (TA as per primer) for 15 s, and elongation at 72°C for 10 s. Subsequent melt curve analysis was performed by heating to 95°C for 10 s, cooling to 65°C for 1 min, and heating to 97°C. Primers sequences and accession numbers are outlined in Table 3. All data for qRT-PCR, where relative expression of each gene was calculated relative to β-actin as a housekeeping gene.

**Table 3:** Primers utilized for quantitative real time-PCR.

Gene name	Primers		Annealing Temp (°C)	Reference
IL-6	Fwd	CAGGACGAGATGTGCAAGAA	64	Brisbin et al. (2011)
	Rev	TAGCACAGAGACTCGACGTT		
IL-8	Fwd	CCAAGCACACCTCTCTTCCA	60	St Paul et al. (2012a)
	Rev	GCAAGGTAGGACGCTGGTAA		
IL-10	Fwd	AGCAGATCAAGGAGACGTTC	60	Brisbin et al. (2010)
	Rev	ATCAGCAGGTACTCCTCGAT		
IL-12p40	Fwd	TTGCCGAAGAGCACCAGCCG	60	Brisbin et al. (2010)
	Rev	CGGTGTGCTCCAGGTCTTGGG		
IL-13	Fwd	ACTTGTCCAAGCTGAAGCTGTC	64	Brisbin et al. (2011)
	Rev	TCTTGCAGTCGGTCATGTTGTC		
IL-17	Fwd	TATCAGCAAACGCTCACTGG	60	St Paul et al. (2012a)
	Rev	AGTTCACGCACCTGGAATG		
IL-18	Fwd	GAAACGTCAATAGCCAGTTGC	60	Brisbin et al. (2010)
	Rev	TCCCATGCTCTTTCTCACAACA		
IL-22	Fwd	TCAACTTCCAGCAGCCCTACAT	60	Kim et al. (2012)
	Rev	TGATCTGAGAGCCTGGCCATT		
CD40	Fwd	CCTGGTGATGCTGTGAATTG	60	St Paul et al. (2012b)
	Rev	CTTCTGTGTCGTTGCATTCAG		
CTLA-4	Fwd	CAAGATGGAGCGGATGTACC	55	Parvizi et al. (2010)
	Rev	TGGCTGAGATGATGATGCTG		
IFN-γ	Fwd	ACACTGACAAGTCAAAGCCGCACA	60	Brisbin et al. (2010)
	Rev	AGTCGTTCATCGGGAGCTTGGC		
TGF-β4	Fwd	CGGCCGACGATGAGTGGCTC	60	Brisbin et al. (2010)
	Rev	CGGGGCCCATCTCACAGGGA		
β-Actin	Fwd	CAACACAGTGCTGTCTGGTGGTA	58	Brisbin et al. (2011)
	Rev	ATCGTACTCCTGCTTGCTGATCC		
Universal	Fwd	AAACTCAAAKGAATTGACGG	61	Bacchetti De Gregoris et al. (2011)
	Rev	CTCACRRCACGAGCTGAC		
*Actinobacteria*	Fwd	TACGGCCGCAAGGCTA	61	Bacchetti De Gregoris et al. (2011)
	Rev	TCRTCCCCACCTTCCTCCG		
*Firmicutes*	Fwd	TGAAACTYAAAGGAATTGACG	61	Bacchetti De Gregoris et al. (2011)
	Rev	ACCATGCACCACCTGTC		
*Bacteroides*	Fwd	CRAACAGGATTAGATACCCT	61	Bacchetti De Gregoris et al. (2011)
	Rev	GGTAAGGTTCCTCGCGTAT		
*γ-proteobacteria*	Fwd	TCGTCAGCTCGTGTYGTGA	61	Bacchetti De Gregoris et al. (2011)
	Rev	CGTAAGGGCCATGATG		
*Lactobacillaceae*	Fwd	AGCAGTAGGGAATCTTCCA	61	Bacchetti De Gregoris et al. (2011)
	Rev	CACCGCTACACATGGAG		

### 2.8 Bacterial DNA extraction

Duodenum, jejunum and ileum intestinal microbial genomic DNA extraction was performed using QIAamp® Fast DNA Stool Mini Kit (Qiagen, Canada) as recommended by the manufacturer. Briefly, 1 mL of InhibitEX buffer was added to 200 mg of duodenum, jejunum and ileum content. After a brief vortex (1 min) and centrifugation (20,000 × g for 1 min), supernatants were collected and added to tubes containing 25 μL of Proteinase K. AL buffer was added to the mixtures and incubated for 10 min at 70°C. Following the addition of 600 μL of 100% Ethanol, lysate-mixtures were briefly vortexed (1 min). The lysate-mixtures were applied to the spin columns, centrifuged (20,000 × g for 1 min) and washed with AW buffers in 2 steps. Eluted DNA (20,000 × g for 1 min) were quantified using a NanoDrop® ND-1000 spectrophotometer (NanoDrop Technologies, Wilmington, DE) and adjusted to 40 ng/μl for SYBR green qRT-PCR. Plates were read and analyzed by Light Cycler 480 II (Roche Diagnostics GmbH Mannheim, Germany). Total bacterial population was determined relative to the universal primers over control samples. Primer sequences and annealing temperatures are outlined in Table 2.

### 2.9 Statistical analysis

Graph Pad Prism 8 for windows was utilized to generate graphs and perform statistical analysis. All data are presented as mean + SD and analyzed by Wilcoxon and Mann Whitney non-parametric as well as two-way ANOVA was used to test significance. Results were considered statistically significant at *p* < 0.05 (*).

## 3 Results

### 3.1 Oral inoculation of r*L. lactis* reduces necrotic enteritis lesions

**Figure 1:**
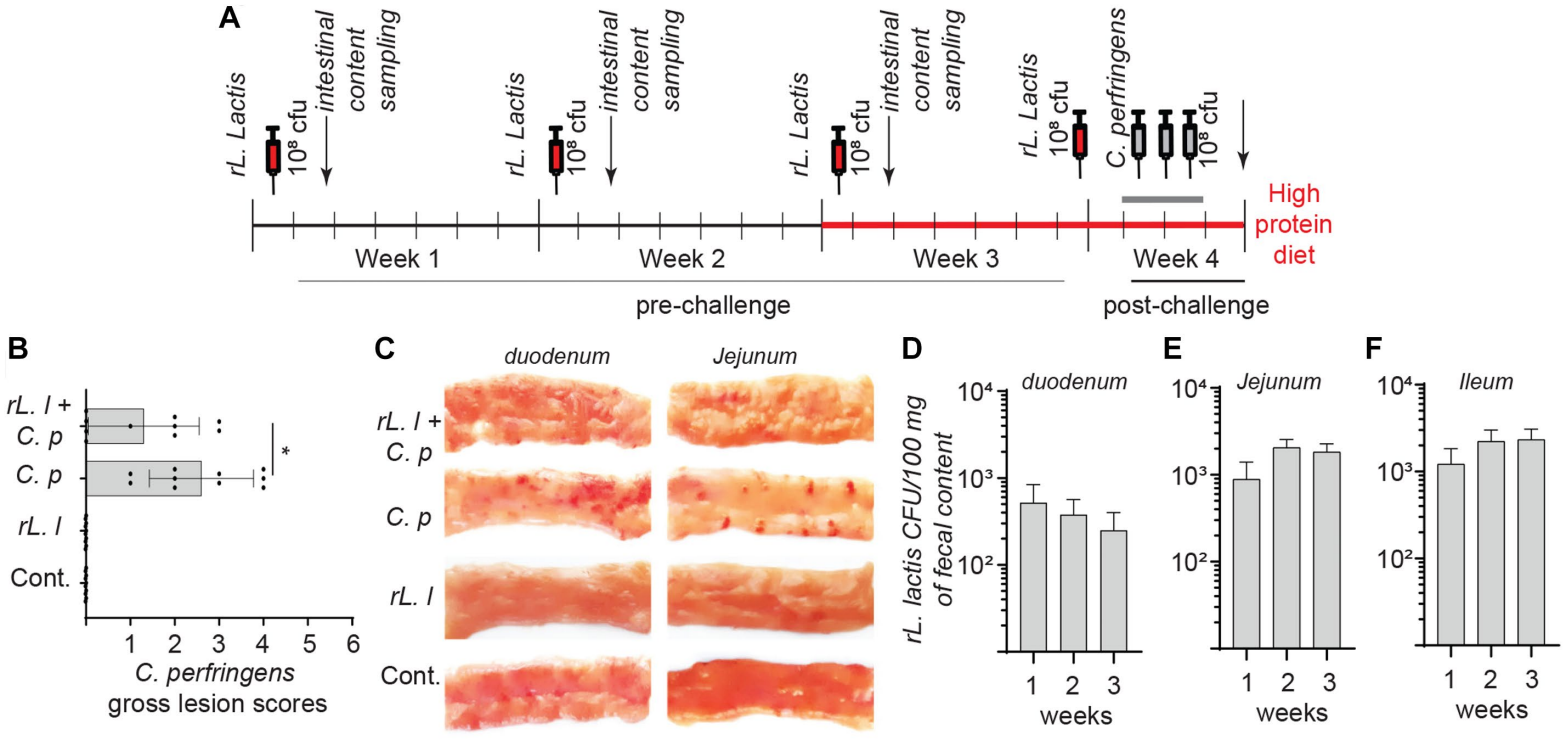
Necrotic lesion in *C. perfringens* infected boiler chickens. Schematic representation of experimental timeline **(A)** whereby 60 broiler chickens were orally administered with a recombinant *L. lactis* (1.0 × 10^8^ CFU) or PBS on day 1, 7, 14 and 21 days of age. Starting week 4 (day 21), 30 broiler chickens were orally infected with *C. perfringens* (3.0 × 10^8^ CFU) twice daily for 3 days. All chickens were sacrificed (*n* = 10 per group) on day 24 and **(B)** necrotic lesion were assessed in all groups and **(C)** a representative image of the necrotic lesion is shown. Presence of recombinant *L. lactis* two-days post inoculation (*n* = 6 weekly per group) was confirmed by serial dilution of intestinal contents collected from **(D)** duodenum, **(E)** jejunum and **(F)** ileum segments. The results are shown as mean ± SD. Non-parametric Wilcoxon tests (Mann–Whitney) was used to test significance. *(*p* ≤ 0.05) indicates a statistically significant result. rL. L; r*L. lactis*, C.P; *C. perfringens*.

*Lactococcus lactis* bacteria were transformed with the pNZ8142 plasmid (r*L. lactis*) to enable detection and enumeration in intestinal contents post inoculation. The experimental timeline is illustrated in Figure 1A. The result demonstrates that chickens infected with *C. perfringens* had lower NE lesions if they had been orally inoculated with a r*L. lactis* (10*8 CFU; *n* = 10) compared to the PBS-treated infected group (*n* = 10) (Figure 1B). Lesion scoring, as listed in Table 1, was performed from the proximal end to the distal end of the small intestine (Figure 1C). Inoculation of r*L. lactis* prior to infection with *C. perfringens* inhibited fibrin formation, necrotic foci and ulcers. However, erythematous mucosa was still evident in these birds. It should be noted that chickens receiving r*L. lactis* or PBS without infection with *C. perfringens* showed no intestinal lesions. The quantification of r*L. lactis* in intestinal contents, collected from at least 6 chicks from each group (24 h post oral gavage), demonstrated that r*L. lactis* pass through and can survive in the medial duodenum (Figure 1D), jejunum (Figure 1E) and ileum (Figure 1F). Positive bacterial colonies were considered as the *rL. lactis* carrying the pNZ8124 plasmid providing resistance to chloramphenicol. Taken together, the results indicate that the presence of r*L. lactis* in the intestinal contents is associated with a reduction in necrotic lesions induced by *C. perfringens*.

### 3.2 r*L. lactis* treatment alters microbial genome content in chicken intestine

**Figure 2:**
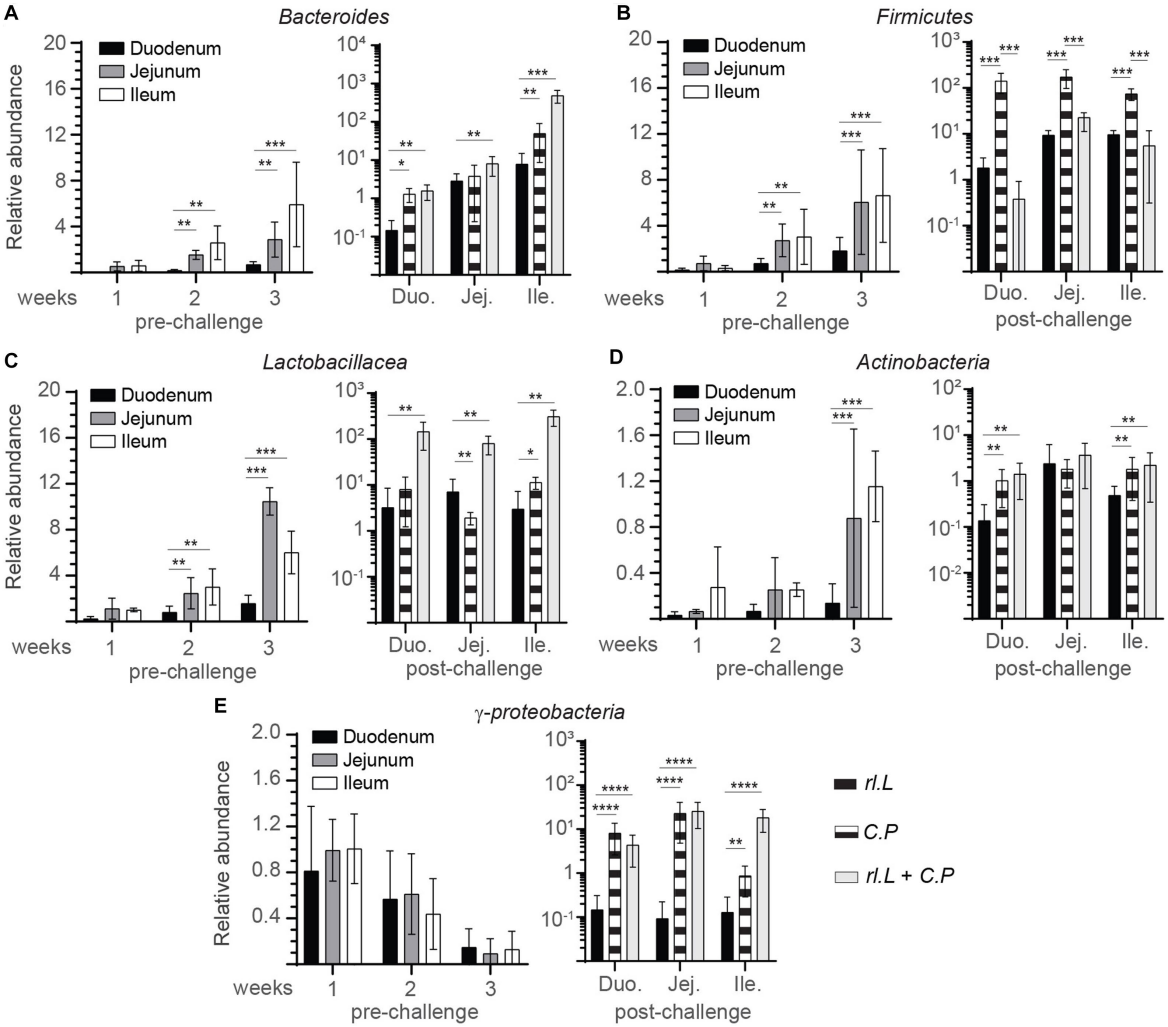
Changes in intestinal microbe genomic content as a result of r*L. lactis* inoculation. Weekly relative abundance of **(A)** Bacteroidota, **(B)** Bacillota, **(C)** Lactobacillaceae, **(D)** Actinomycetota, **(E)** Pseudomonadota *to universal primers over control* were assessed in intestinal contents collected 2 days post r*L. lactis* inoculation from the medial duodenum, jejunum and ileum intestinal segments prior to (*n* = 6 per group) and post *C. perfringens* infection (*n* = 10 per group) over control/PBS inoculated chickens. DNA extracted from 100 mg of intestinal contents were utilized for real-time PCR. The results are shown as mean ± SD. Non-parametric Wilcoxon tests (Mann–Whitney) was used to test significance. *(*p* ≤ 0.05), **(*p* < 0.01), and ****(*p* < 0.001) indicates a statistically significant result. Duo, duodenum; Jej, jejunum; Ile, ileum; Cont., control/PBS; rL. L, r*L. lactis*; C.P, *C. perfringens*.

To examine any possible association between necrotic lesions and the abundance of microbial groups, intestinal contents (*n* = 6 per time points) were analyzed to determine phylogenetic relations in the r*L. lactis* treated chickens compared to control/PBS treated chickens prior to and post *C. perfringens* infection (Figure 2). Temporal changes in microbial relative content were used to estimate the abundance of specific bacterial phylum: Bacillota *(Firmicutes)*, Bacteroidota *(Bacteroides)*, Lactobacillaceae *(Lactobacillus)*, Pseudomonadota *(γ-proteobacteria)* and Actinomycetota *(Actinobacteria)* to universal primers at 36 h post oral gavage with *rL. lactis*. The assessment of intestinal contents indicated that a diverse microbial community was established and could be modified by r*L. lactis* treatment. The results demonstrate site specific and temporal increases (*p* ≤ 0.01) in the Bacteroidota, Bacillota, and Lactobacillaceae prior to infection. There was a significant increase in the relative abundance of Bacteroidota (Figure 2A), Bacillota (Figure 2B), and Lactobacillaceae (Figure 2C) in jejunum (*p* ≤ 0.01) and ileum (*p* ≤ 0.01) at week 2 and 3 compared to that observed in duodenum. However, Actinomycetota (Figure 2D) relative number was significantly (*p* ≤ 0.005) more abundant in jejunum and ileum content at week 3 in comparison to that in duodenum. No changes in Pseudomonadota were observed after *rL. lactis* treatment alone in comparison to the control group (Figure 2E). Post *C. perfringens* infection, treatment with *rL. lactis* led to a decrease in the Bacillota but an increase in Bacteroidota, Lactobacillaceae, Pseudomonadota and Actinomycetota. The results also demonstrate that infection alone with *C. perfringens* (day 24) led to a significant (*p* ≤ 0.01) increase in duodenum, jejunum and ileum abundance of the Bacillota (Figure 2B), Actinomycetota (Figure 2D) and Pseudomonadota (Figure 2E). However, prior treatment with *rL. lactis* and subsequent infection with *C. perfringens* (day 24) resulted in a significant (*p* ≤ 0.005) decrease in duodenum, jejunum and ileum abundance of the Bacillota (Figure 2B). In contrast, prior treatment with *rL. lactis* and subsequent infection with *C. perfringens* (day 24) resulted in a significant (*p* ≤ 0.005) increase in duodenum, jejunum and ileum intestinal content of the Bacteroidota (Figure 2A), Lactobacillaceae (Figure 2C), Actinomycetota (Figure 2D) and Pseudomonadota (Figure 2E).

### 3.3 Dynamics of induction of pro-inflammatory cytokines and mucosal innate immune cells

**Figure 3:**
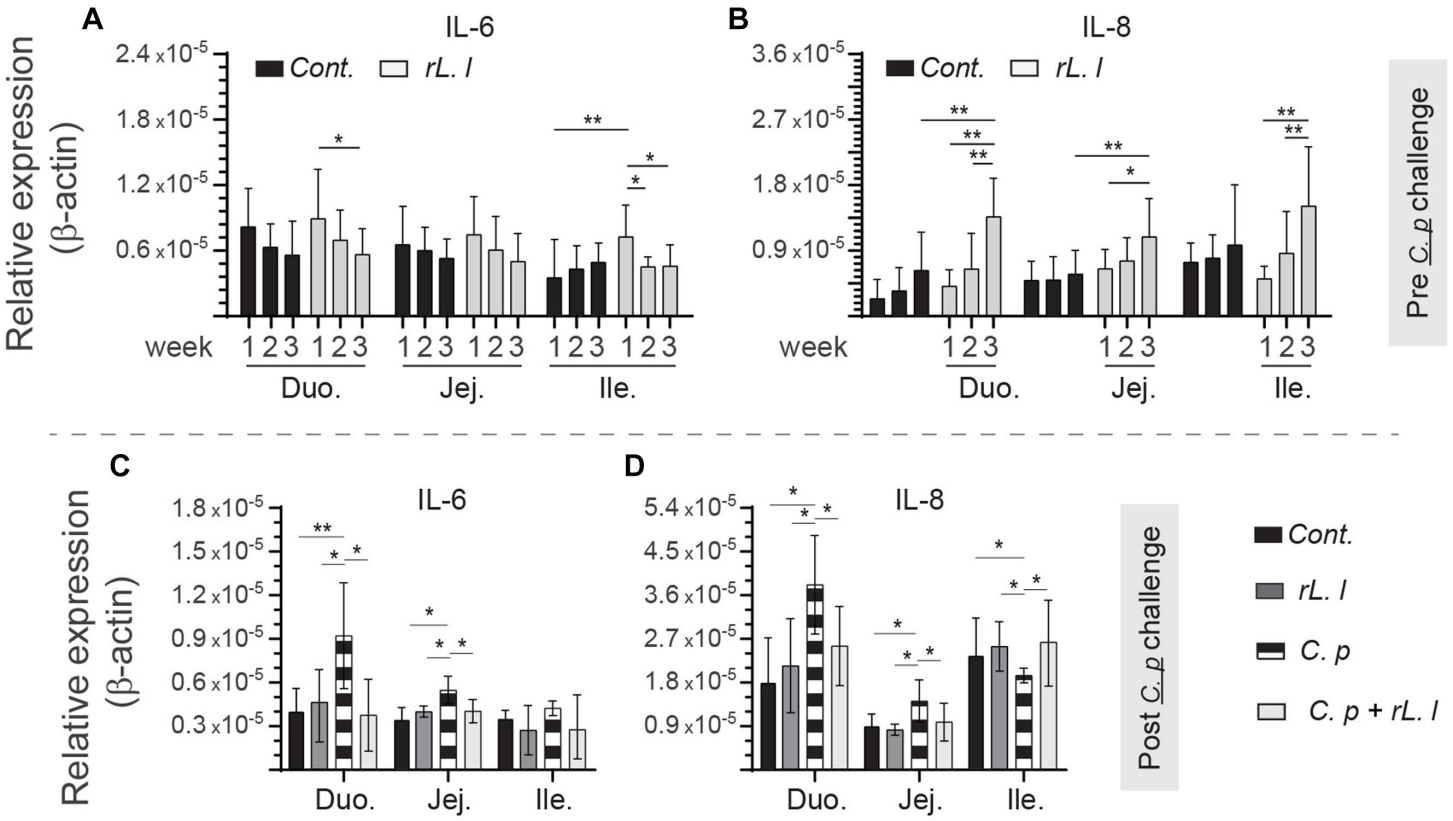
Temporal changes in intestinal IL-6, and IL-8. Weekly relative **(A,C)** *IL-6*, and **(B,D)** *IL-8* to β-actin mRNA transcripts in medial duodenum, jejunum and ileum intestinal segments from *rL. lactis* treated chickens **(A,B)** prior to (*n* = 6 per group) and **(C,D)** post *C. perfringens* infection (*n* = 10 per group) over β-actin in control/PBS inoculated chickens. The results are shown as mean ± SD. Non-parametric Wilcoxon tests (Mann–Whitney) or two-way ANOVA was used to test significance. *(*p* ≤ 0.05), and **(*p* ≤ 0.01) indicates a statistically significant result. Duo, duodenum; Jej, jejunum; Ile, ileum; Cont., control/PBS; rL. L, r*L. Lactis*; C.P, *C. perfringens*.

To identify the effects of *rL. lactis* treatment on modulation of mucosal immune parameters, we analyzed induction of innate responses, such as temporal expression of IL-6, and IL-8 to β-actin as well as frequency of intestinal mononuclear phagocytes. Treatment with *rL. lactis* prior to infection with *C. perfringens* led to a time- and site-specific dependent increase in expression of IL-6 (Figure 3A), and IL-8 (Figure 3B). Over time (week 1 vs. week 3), IL-6 expression was significantly decreased in the duodenum and jejunum of *rL. lactis* treated chickens (Figure 3A). There was a significant (*p* ≤ 0.05) decrease in expression of IL-6 (Figure 3C), and IL-8 (Figure 3D) in the duodenum and jejunum of chickens that were pre-treated with *rL. lactis* and infected with *C. perfringens* in comparison to *C. perfringens* only infected chickens. In contrast, infection with *C. perfringens* led to a significant increase (*p* ≤ 0.05) in expression of IL-8 (Figure 3D) when comparing to *rL. lactis* treated only and *rL. lactis* and *C. perfringens* infected groups in the ileum.

**Figure 4:**
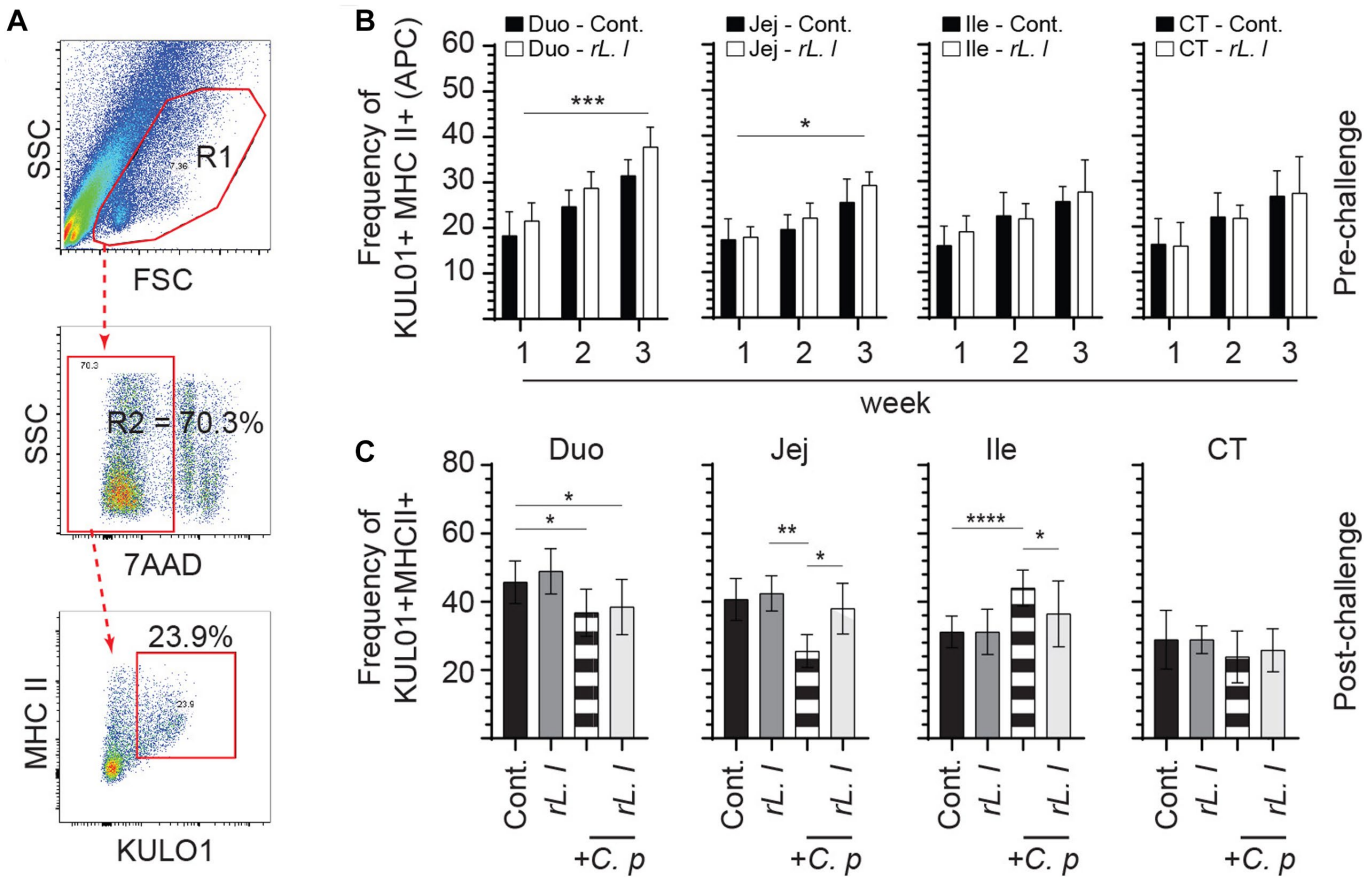
Changes in the frequency of intestinal mononuclear phagocytes. The frequency of duodenum, jejunum, ileum and cecal tonsils mononuclear cells were assessed in r*L. lactis* inoculated chickens prior to and post challenge with PBS or *C. perfringens* in comparison *C. perfringens* infected only and control chickens (*n* = 6 per group). **(A)** Dot plot demonstrates gating strategy for detection of macrophages (KUL01 + MHC-II+) are shown. The weekly frequency (7AAD-; dead cell exclusion) of KUL01 + MHC-II+ in the **(B)** pre-challenge **(C)** and post *C. perfringens* challenge are presented. Non-parametric Wilcoxon tests (Mann–Whitney) or two-way ANOVA was used to test significance with the results shown as mean ± SD. *(*p* ≤ 0.05), **(*p* ≤ 0.01), ***(*p* ≤ 0.005), and ****(*p* ≤ 0.001) indicates a statistically significant result. The mean ± SD value are shown in weekly samples collected from six individual birds for each group. Duo, duodenum; Jej, jejunum; Ile, ileum; CT, cecal tonsils; Cont., control; rL. L, r*L. Lactis*; C.P, *C. perfringens.*

As IL-6 and IL-8 can be produced by mononuclear phagocytes, we analyzed the frequencies of Kul01 + MHC-II+ cells in different experimental groups using flow cytometry (Figure 4A). The results demonstrate a significant increase in frequency of the Kul01 + MHC-II+ cells, following *rL. lactis* treatment, in the medial duodenum (*p* ≤ 0.0001), and jejunum (*p* ≤ 0.05) but not in the ileum and cecal tonsil when compared to the control chickens (Figure 4B). Infection with *C. perfringens* led to a significant decrease in frequency of the Kul01 + MHC-II+ cells in duodenum (*p* ≤ 0.05) and jejunum (*p* ≤ 0.05) compared to the control or the *rL. lactis* treated and infected chickens (Figure 4C). There was a significant (*p* ≤ 0.0001) increase in frequencies of Kul01 + MHCII+ cells in ileum of the *C. perfringens* infected chickens compared to the control or the *rL. lactis* treated only groups (Figure 4C). The increase in mucosal Kul01 + MHC-II+ cells was sustained post infection with *C. perfringens* (Figure 4C).

### 3.4 Temporal expression profile of cytokines that modulate T cell function

**Figure 5:**
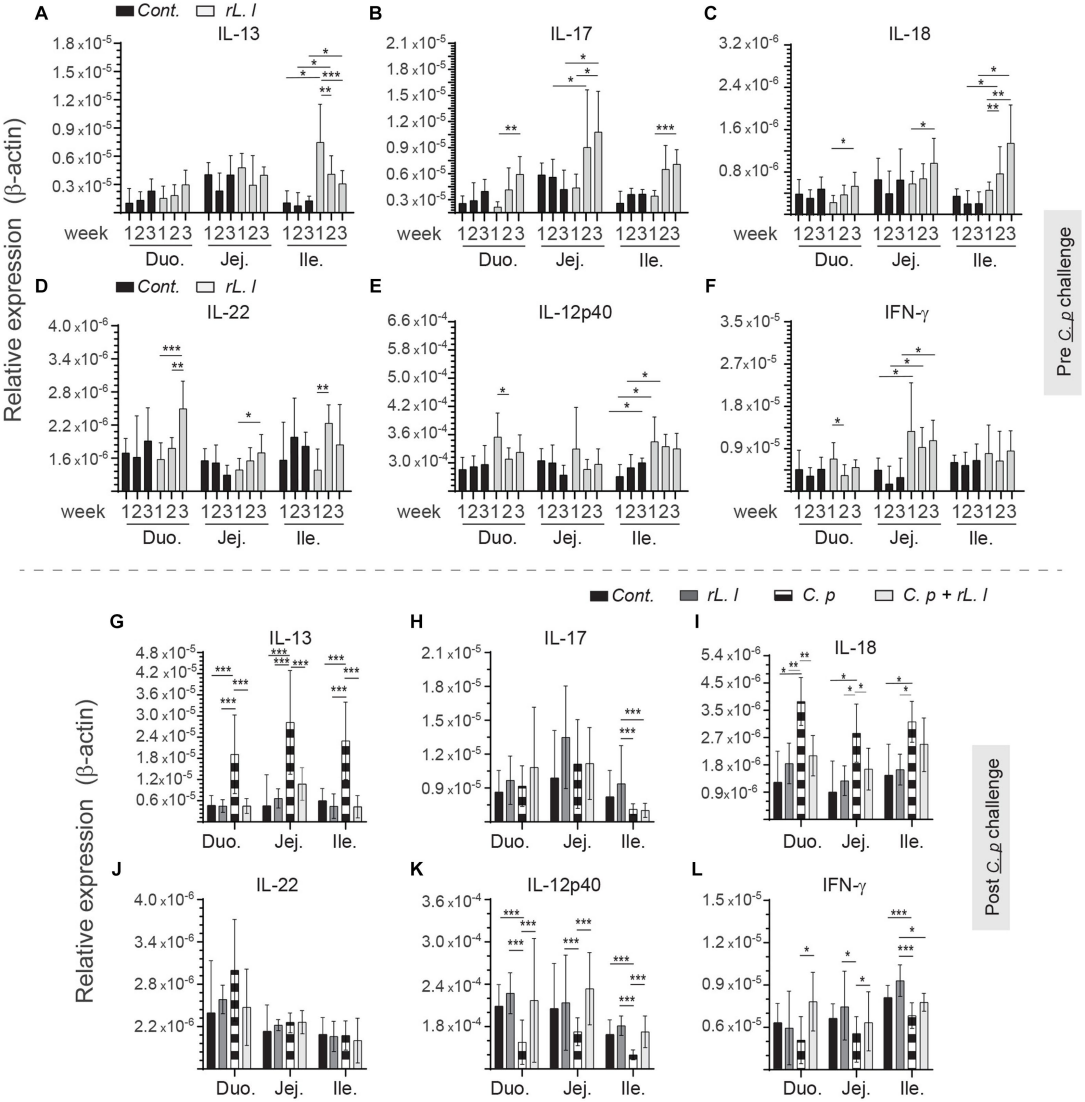
Temporal changes in intestinal IL-13, IL-17, IL-18 IL-22, IL-12p40 and IFN-γ. Weekly relative **(A,G)** *IL-13*, **(B,H)** *IL-17*, **(C,I)** *IL-18*, **(D,J)** *IL-22*, **(E,K)** *IL-12p40* and **(F,L)** *IFN-γ* to β-actin mRNA transcripts in medial duodenum, jejunum and ileum intestinal segments from *rL. Lactis* treated chickens **(A–F)** prior to (*n* = 6 per group) and **(G–L)** post *C. perfringens* infection (*n* = 10 per group) over β-actin in control/PBS inoculated chickens. The results are shown as mean ± SD. Non-parametric Wilcoxon tests (Mann–Whitney) or two-way ANOVA was used to test significance. *(*p* ≤ 0.05) **(*p* ≤ 0.01) ***(*p* ≤ 0.005) indicates a statistically significant result. Duo, duodenum; Jej, jejunum; Ile, ileum; Cont., control/PBS; rL. L, r*L. lactis*; C.P, *C. perfringens.*

*rL. lactis* treatment prior to infection with *C. perfringens* led to temporal and spatial changes in expression of IL-13 (Figure 5A), IL-17 (Figure 5B), IL-18 (Figure 5C), IL-22 (Figure 5D), IL-12p40 (Figure 5E) and IFN-γ (Figure 5F) to β-actin from week 1 onwards in all sites along the intestine. IL-17 (Figure 5B), and IFN-γ (Figure 5F) mRNA transcripts were significantly (*p* ≤ 0.01) upregulated in the jejunum of *rL. lactis* treated group but not in the duodenum and ileum. In contrast, IL-13 (Figure 5A) and IL-18 (Figure 5C) mRNA transcripts were significantly (*p* ≤ 0.05) upregulated in the ileum of *rL. lactis* treated group. There was a significant (*p* ≤ 0.05) decrease in expression of intestinal IL-13 (Figure 5G) and IL-18 (Figure 3I) in chickens that were pre-treated with *rL. lactis* and subsequently infected with *C. perfringens* in comparison with *C. perfringens* only infected chickens. Post- *C. perfringens* infection, there was a significant increase in intestinal IL-13 (*p* ≤ 0.001) (Figure 5G) and IL-18 (*p* ≤ 0.05) (Figure 5I) expression in both *rL. lactis* treated only and *rL. lactis* treated and subsequently *C. perfringens* infected chickens compared to control chickens. Expression of IL-17 was significantly decreased (*p* ≤ 0.001) in the ileum only and not in duodenum and jejunum in both *rL. lactis* treated only and *rL. lactis* treated and subsequently *C. perfringens* infected chickens compared to control chickens (Figure 5H). Both IL-12p40 (Figure 5K) and IFN-γ (Figure 5L) intestinal mRNA transcripts were significantly increased (*p* ≤ 0.001) in *rL. lactis* treated and *C. perfringens* infected chickens when compared to *C. perfringens* only infected chickens. No change in IL-22 (Figure 5J) was observed post-infection with *C. perfringens*.

### 3.5 Challenge with *Clostridium perfringens* led to an increase in intestinal γδ T cells

**Figure 6:**
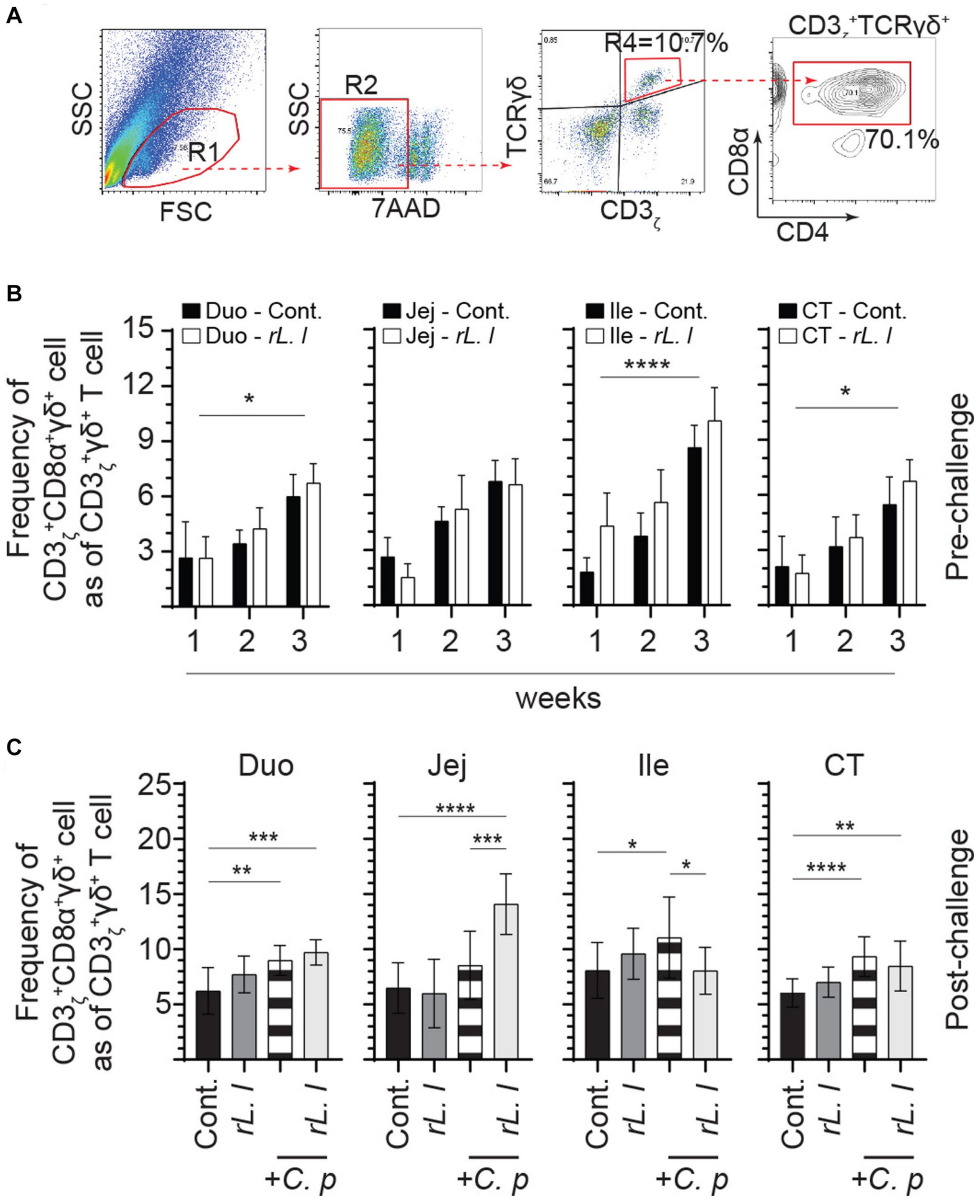
Increase in the frequency of intestinal γδ T cells in response to r*L. lactis* inoculation. The frequency of duodenum, jejunum, ileum and cecal tonsils mononuclear cells were assessed in r*L. lactis* inoculated chickens prior to and post challenge with PBS or *C. perfringens* in comparison to *C. perfringens* infected only and control chickens (*n* = 6 per group). **(A)** Pseudocolor plot demonstrates gating strategy for detection of CD3 + CD8α + γδ T cells are shown. The weekly frequency (7AAD-; dead cell exclusion) of CD3 + CD8α + γδ T in γδ T cells **(B)** pre-challenge **(C)** and post *C. perfringens* challenge are presented. Non-parametric Wilcoxon tests (Mann–Whitney) or two-way ANOVA was used to test significance with the results shown as mean ± SD. *(*p* ≤ 0.05), **(*p* ≤ 0.01), ***(*p* ≤ 0.005), and ****(*p* ≤ 0.001) indicates a statistically significant result. The mean ± SD value are shown in weekly samples collected from six individual birds for each group. Duo, duodenum; Jej, jejunum; Ile, ileum; CT, cecal tonsils; Cont., control; rL. L, r*L. lactis*; C.P, *C. perfringens.*

Alterations in intestinal cytokines can affect intestinal cellular composition, such as abundance of T cells. FACS analysis was used to characterize intestinal mononuclear cells along the intestinal tissue (Figure 6A). The frequencies of CD3ζ + CD8α + γδ T cells within CD3ζ + γδ T cell population are presented. In control chickens, the ileum was found to have higher frequencies of CD3ζ + CD8α + γδ T cells in comparison with the duodenum, jejunum and cecal tonsils. The results also demonstrate that treatment with *rL. lactis* led to a significant increase in the frequency of CD3ζ + CD8α + γδ T cells in the duodenum (*p* ≤ 0.05), ileum (*p* ≤ 0.0001) and cecal tonsils (*p* ≤ 0.05) as assessed by two-way ANOVA when compared to control chickens (Figure 6B). Infection with *C. perfringens* also led to a significant increase in the frequency of CD3ζ + CD8α + γδ T cells in duodenum (*p* ≤ 0.01), ileum (*p* ≤ 0.05) and cecal tonsils (*p* ≤ 0.005) (Figure 6C). Similarly, *rL. lactis* treatment followed by infection with *C. perfringens* increased the frequency of these cells in duodenum (*p* ≤ 0.005), jejunum (*p* ≤ 0.0001) and cecal tonsils (*p* ≤ 0.01) CD3ζ + CD8α + γδ T cells when compared to control chickens (Figure 6C).

### 3.6 Temporal and site-specific changes in adaptive immune system cells (αβ T and B cells)

**Figure 7:**
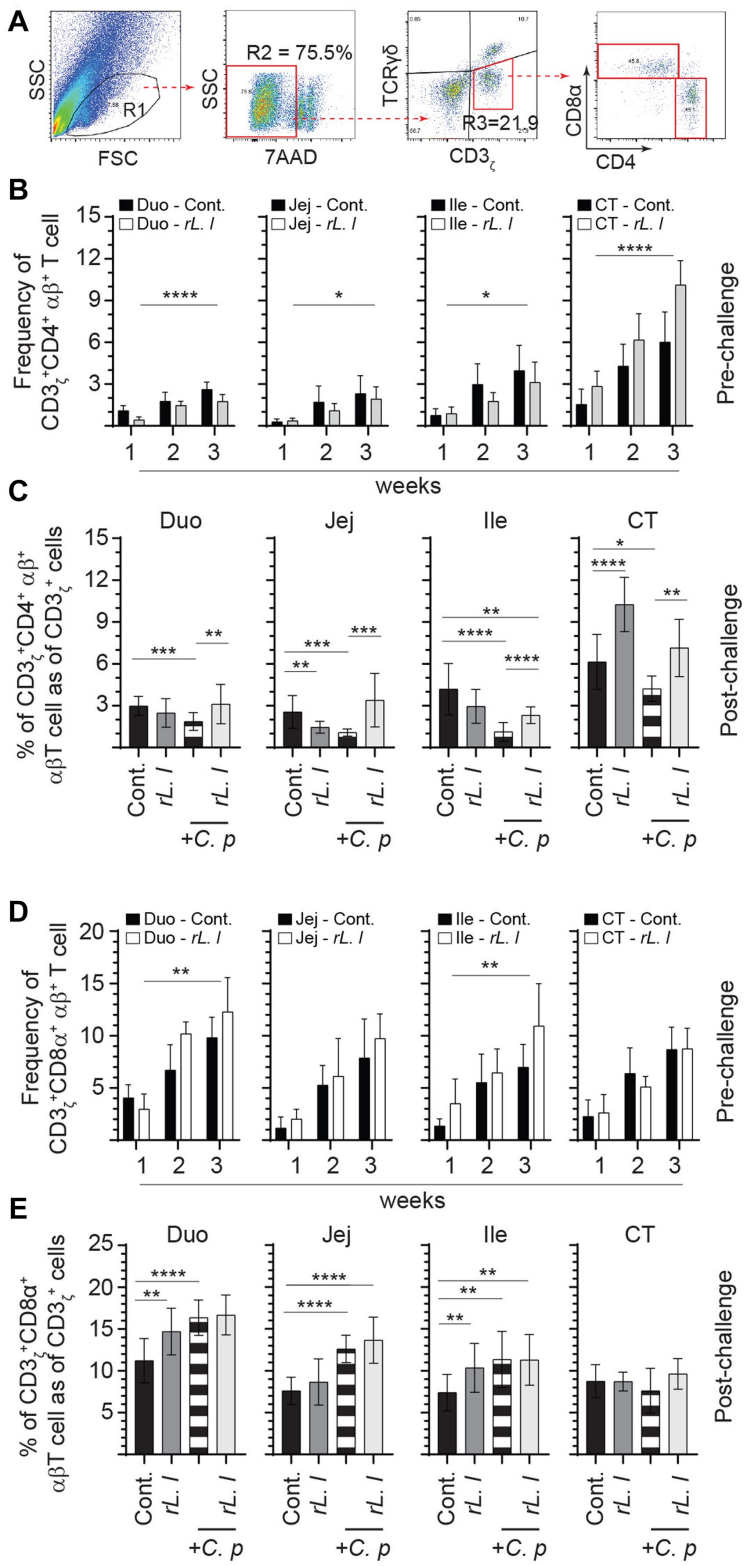
Differential changes in the frequency of intestinal αβ T cells subsets in response to r*L. lactis* inoculation. The frequency of duodenum, jejunum, ileum and cecal tonsils mononuclear cells were assessed in r*L. lactis* inoculated chickens prior to and post challenge with PBS or *C. perfringens* in comparison to *C. perfringens* infected only and control chickens (*n* = 6 per group). **(A)** Pseudocolor plot demonstrates gating strategy for detection of CD3+ αβ T cells that are **(B,C)** CD4+ or **(D,E)** CD8α + are shown. The weekly frequency (7AAD-; dead cell exclusion) of CD3 + CD4+ and CD3 + CD8α + αβ T in CD3+ αβ T cells **(B,D)** pre-challenge **(C,E)** and post *C. perfringens* challenge are presented. Non-parametric Wilcoxon tests (Mann–Whitney) or two-way ANOVA was used to test significance with the results shown as mean ± SD. *(*p* ≤ 0.05), **(*p* ≤ 0.01), and ***(*p* ≤ 0.0005) and ****(*p* ≤ 0.0001) indicates a statistically significant difference compared to control. The mean ± SD value are shown in weekly samples collected from six individual birds for each group. Duo, duodenum; Jej, jejunum; Ile, ileum; CT, cecal tonsils; Cont., control; rL. L, r*L. lactis*; C.P, *C. perfringens.*

The frequencies of αβ T cells were also assessed by FACS analysis. A negative gating strategy, CD3ζ + γδ- T cells, was used to identify CD3ζ + αβ + T cells that are either express CD4+ or CD8α + (Figure 7A). CD3ζ + CD8α + αβ T cells (Figure 7D) made up the majority of the αβ T cells and were more abundant (on average 2:1 ratio) than CD3ζ + CD4+ αβ T cells (Figure 7B). The abundance of intestinal tissue CD3ζ + CD8α + αβ T cells was further amplified following a prolonged treatment with *rL. lactis.* The results demonstrate that treatment with *rL. lactis* led to a significant increase in the frequency of CD3ζ + CD8α + αβ T cells in the duodenum (*p* ≤ 0.01) and ileum (*p* ≤ 0.01) when compared to the control chickens (Figure 7D). In contrast, treatment with *rL. lactis* led to a significant decrease in the frequency of intestinal CD3ζ + CD4+ αβ T cell when compared to the control chickens (Figure 7B). Infection with *C. perfringens* also altered the frequency of CD4+ and CD8α + αβ T cells. Infection with *C. perfringens* resulted in a significant decrease in the frequency of CD3ζ + CD4+ αβ T cells across all segments of the intestine (Figure 7C). Conversely, infection significantly increased the frequency of CD3ζ + CD8α + αβ T cells across all segments of the intestine when compared to the *rL. lactis* treated and infected chickens (Figure 7E). However, the combination of *rL. lactis* treatment and subsequent *C. perfringens* infection resulted in the increase of both CD3ζ + CD4+ (Figure 7C) and CD3ζ + CD8α + (Figure 7E) αβ T across the intestine when compared to the infected or control chickens, respectively.

**Figure 8:**
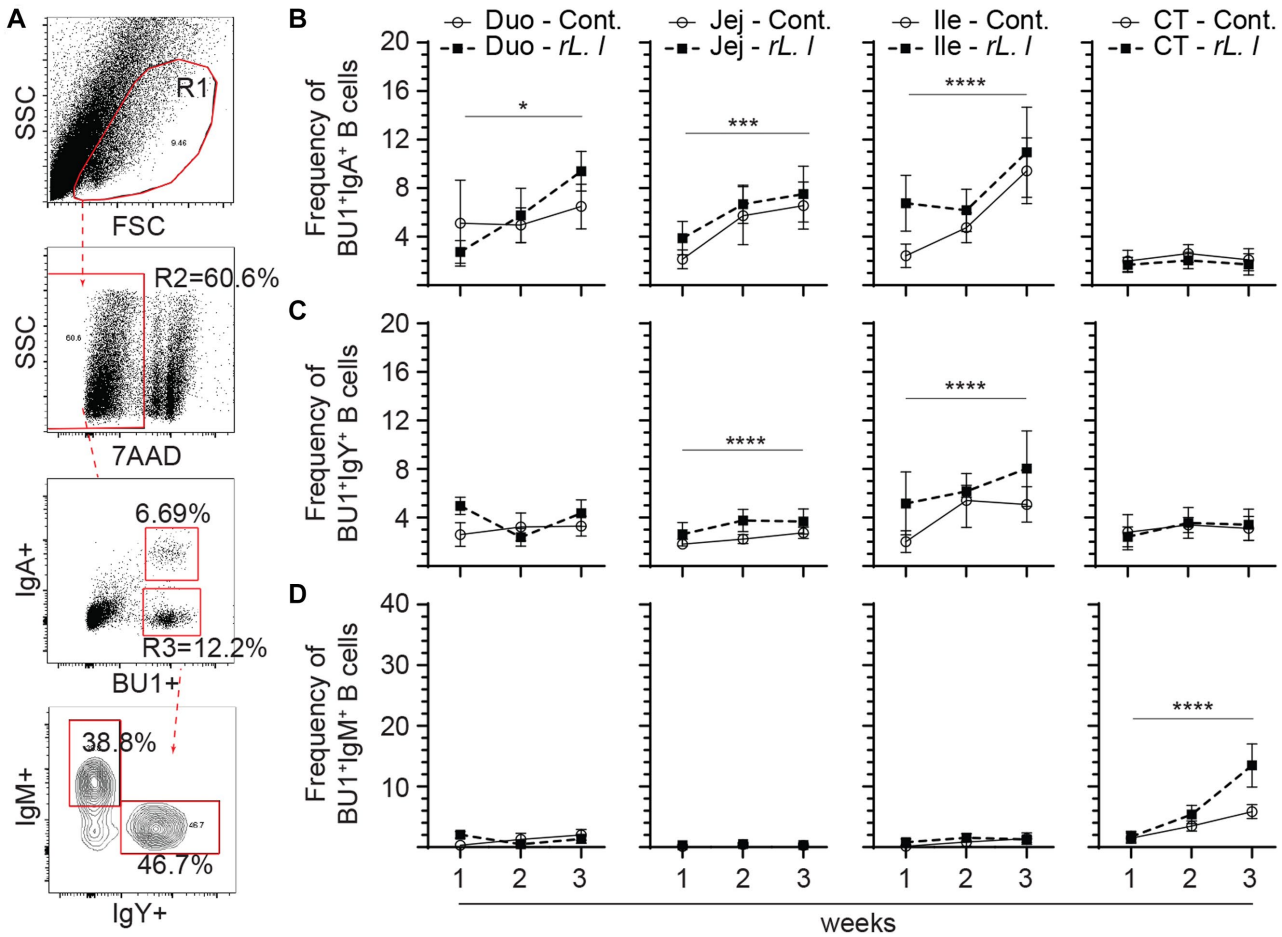
Differential changes in the frequency of intestinal B cell subsets in response to r*L. lactis* inoculation. The frequency of duodenum, jejunum, ileum and cecal tonsils mononuclear cells were assessed in r*L. lactis* inoculated chickens in comparison to control chickens (*n* = 6 per group). **(A)** Dot plot demonstrates gating strategy for detection of Bu1+ B cells and the various subsets; IgA+, IgY+ and IgM+. The weekly frequencies (7AAD-; dead cell exclusion) of Bu1+ B cells that are **(B)** IgA+, **(C)** IgY+ and **(D)** IgM+ pre-challenge are presented. Non-parametric Wilcoxon tests (Mann–Whitney) or two-way ANOVA was used to test significance with the results shown as mean ± SD. *(*p* ≤ 0.05), ***(*p* ≤ 0.0005) and ****(*p* ≤ 0.0001) indicates a statistically significant difference compared to control. The mean ± SD value are shown in weekly samples collected from six individual birds from each group. Duo, duodenum; Jej, jejunum; Ile, ileum; CT, cecal tonsils; Cont., control; rL. L, r*L. lactis*.

In addition to T cells, B cells were also characterized in the present study (Figure 8). As expected, three major B cell subsets expressing either IgM, IgY or IgA immunoglobulins were found in the intestine (Figure 8A). The intestine was mainly populated by IgA+ (Figure 8B) followed by IgY+ B cells (Figure 8C) while IgM+ B cells (Figure 8D) made up a minority portion of duodenum, jejunum and ileum Bu1+ B cells. In contrast, the cecal tonsils are mainly populated (*p* ≤ 0.0001) by IgM+ B cells (Figure 8D) followed by IgY+ (Figure 8C) and IgA+ B cells (Figure 8C). The results demonstrated that repeated treatment with *rL. lactis* over several weeks resulted in the expansion of B cells that are IgA+ within the duodenum (*p* ≤ 0.05), jejunum (*p* ≤ 0.005) and ileum (*p* ≤ 0.0001) and IgY+ within the jejunum (*p* ≤ 0.0001) and ileum (*p* ≤ 0.0001) when compared to control chickens. There were no changes in the frequency of IgM+ B cells within the duodenum, jejunum and ileum as a result of *rL. lactis*.

**Figure 9:**
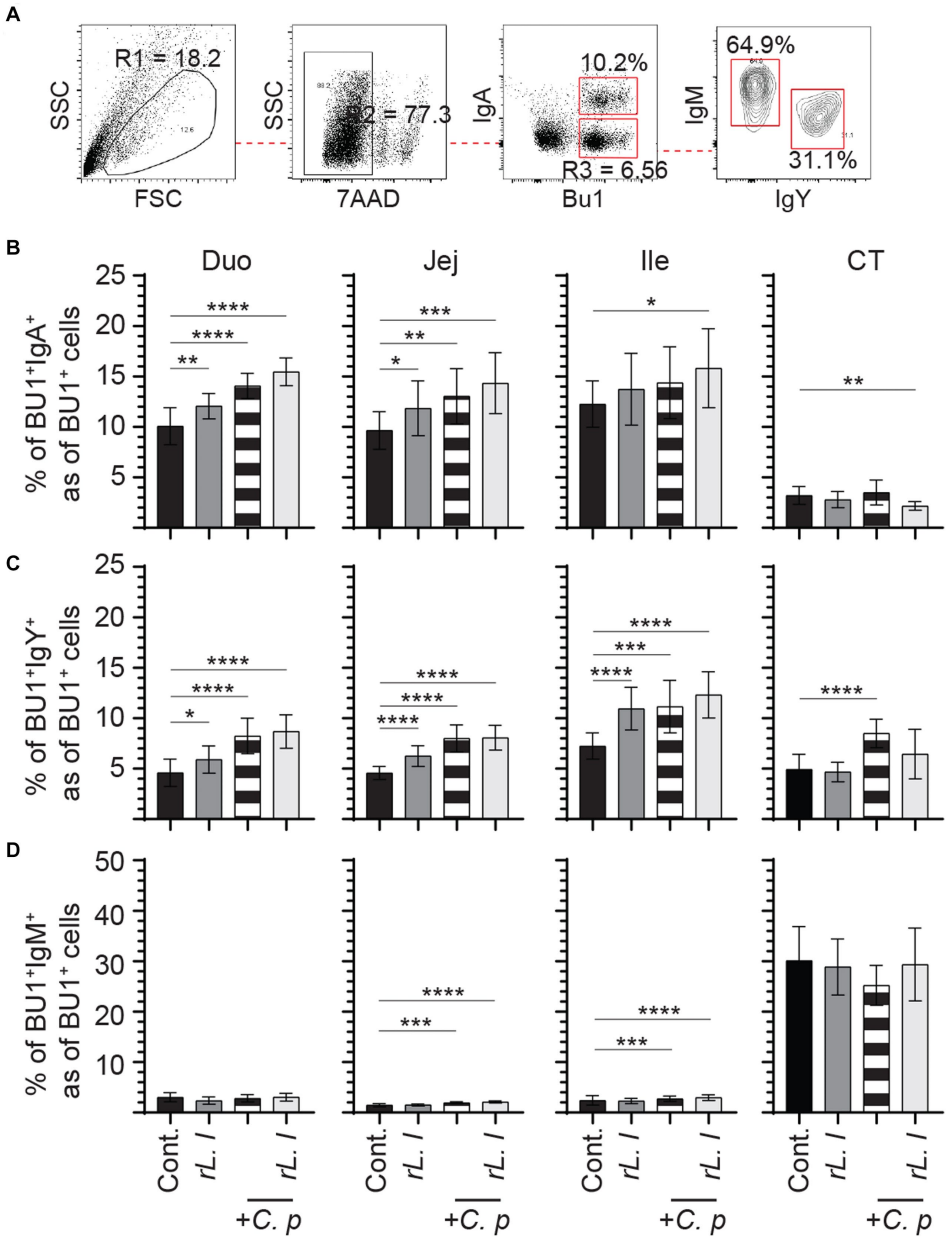
The frequency of intestinal B cell subsets post *C. perfringens* infection. The frequency of duodenum, jejunum, ileum and cecal tonsils mononuclear cells were assessed in r*L. lactis* inoculated chickens prior to and post challenge with PBS or *C. perfringens* in comparison to *C. perfringens* infected only and control chickens (*n* = 6 per group). **(A)** Dot plot demonstrates gating strategy for identification of Bu1+ B cells that are IgA+, IgY+ and IgM+. The weekly frequency (7AAD-; dead cell exclusion) of Bu1+ B cells that are **(B)** IgA+, **(C)** IgY+ and **(D)** IgM+ post *C. perfringens* infection are presented. Non-parametric Wilcoxon tests (Mann–Whitney) was used to test significance with the results shown as mean ± SD. *(*p* ≤ 0.05), **(*p* ≤ 0.01), ***(*p* ≤ 0.0005) and **** (*p* ≤ 0.0001) indicates a statistically significant difference compared to control. The mean ± SD value are shown in weekly samples collected from six individual birds for each group. Duo, duodenum; Jej, jejunum; Ile, ileum; CT, cecal tonsils; Cont., control; rL. L, r*L. lactis*; C.P, *C. perfringens.*

Infection with *C. perfringens* resulted in further expansion of IgA+, IgY+ and IgM+ Bu1+ B cells across the intestine (Figure 9). The results demonstrate that infection with *C. perfringens* led to a significant increase in the frequency of both IgA+ (*p* ≤ 0.0001) (Figure 9B) and IgY+ (*p* ≤ 0.0001) (Figure 9C) Bu1+ B cell within the duodenum, jejunum and cecal tonsils when compared to the control chickens. The combination of prolonged *rL. lactis* pre-treatment followed by infection with *C. perfringens* resulted in a significant increase of both IgA+ (*p* ≤ 0.0001) (Figure 9B) and IgY+ (*p* ≤ 0.0001) (Figure 9C) Bu1+ B cell frequencies within the duodenum, jejunum and ileum when compared to control chickens. The frequency of IgM+ Bu1+ B cell frequency was significantly increased in the jejunum (*p* ≤ 0.005) and ileum (*p* ≤ 0.0001) of chickens that were previously treated with *rL. lactis* and subsequently infected with *C. perfringens* or in *C. perfringens* only infected chickens (Figure 9D).

### 3.7 Temporal expression of molecules associated with immune regulation

**Figure 10:**
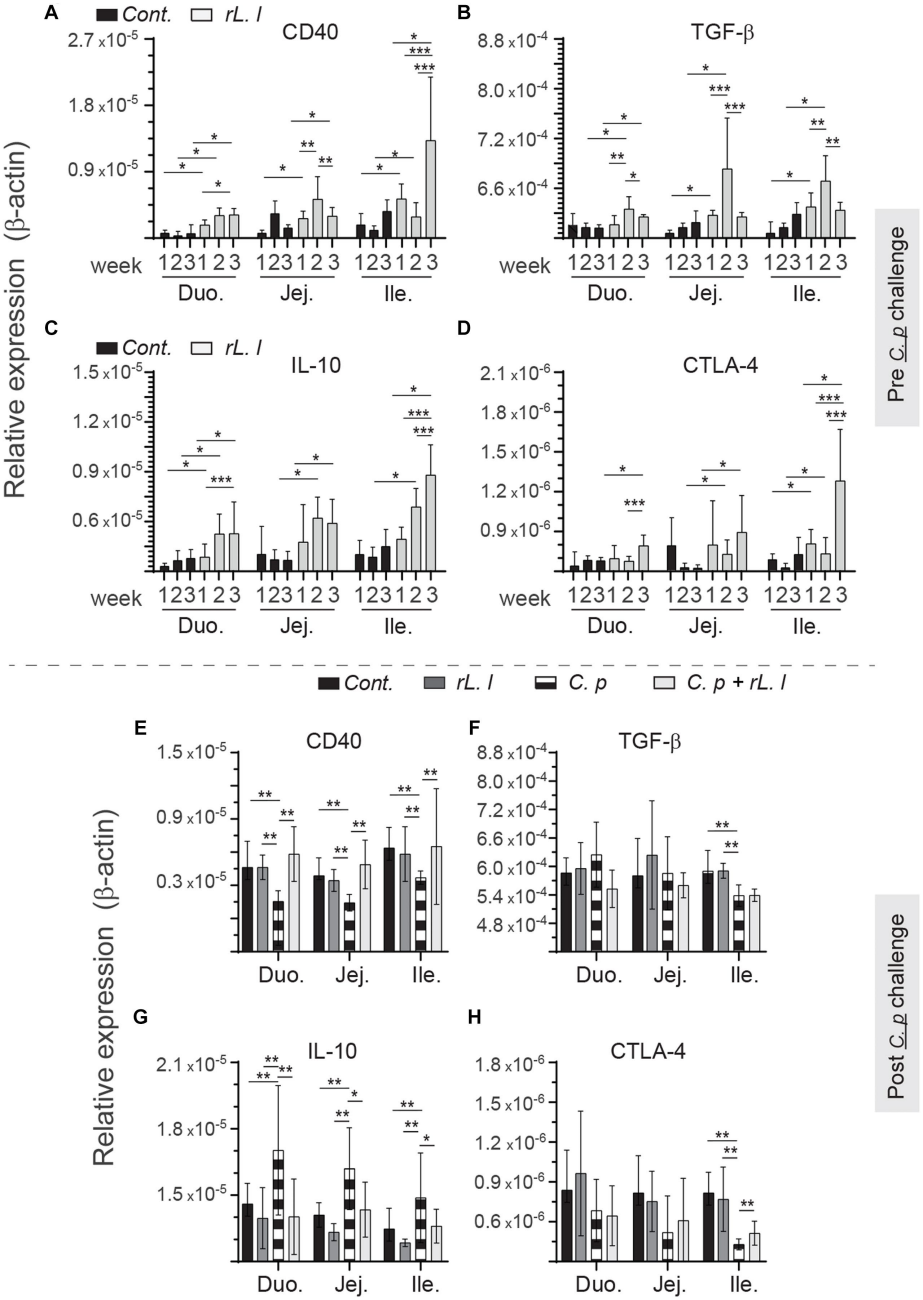
Temporal changes in intestinal CD40, TGF-β, IL-10 and CTLA-4. Weekly relative **(A,F)** *CD40*, **(B,G)** *TGF-β*, **(C,H)** *IL-10* and **(D,I)** *CTLA-4* to β-actin mRNA transcripts in medial duodenum, jejunum and ileum intestinal segments from *rL. Lactis* treated chickens **(A–C)** prior to (*n* = 6 per group) and **(D–F)** post *C. perfringens* infection (*n* = 10 per group) over β-actin in control/PBS inoculated chickens. The results are shown as mean ± SD. Non-parametric Wilcoxon tests (Mann–Whitney) or two-way ANOVA was used to test significance. *(*p* ≤ 0.05) **(*p* ≤ 0.01) ***(*p* ≤ 0.005) indicates a statistically significant result. Duo, duodenum; Jej, jejunum; Ile, ileum; Cont., control/PBS; rL. L, r*L. lactis*; C.P, *C. perfringens.*

To determine the immune regulatory effects of *rL. lactis*, temporal expression of IL-10, TGF-β, CTLA-4 and CD40 was assessed to β-actin (Figure 10). Prior treatment with *rL. lactis* led to an increase in expression of CD40 (Figure 10A), TGF-β (Figure 10B), IL-10 (Figure 10C) and CTLA-4 (Figure 10D) in the duodenum, jejunum and ileum. Infection with *C. perfringens* in those chickens that had prior treatment with *rL. lactis* resulted in a significant (*p* ≤ 0.01) increase in CD40 expression in comparison to *C. perfringens* infected only chickens (Figure 10E). CD40 (Figure 10E) expression levels were significantly (*p* ≤ 0.01) decreased in the duodenum, jejunum and ileum post infection with *C. perfringens*. However, CTLA-4 expression was only decreased in the ileum post infection with *C. perfringens* (Figure 10H). IL-10 mRNA expression level was significantly (*p* ≤ 0.01) decreased in chicken pretreated with *rL. lactis* and subsequently infected with *C. perfringens*, similar to *rL. lactis* treated only chickens, when compared to infected only chickens (Figure 10G). In contrast, infection with *C. perfringens* resulted in a significant (*p* ≤ 0.01) increase in IL-10 mRNA transcripts (Figure 10G) in all intestinal segments studied. No changes in induction of TGF-β mRNA transcripts was observed (Figure 10F).

## 4 Discussion

**Figure 11:**
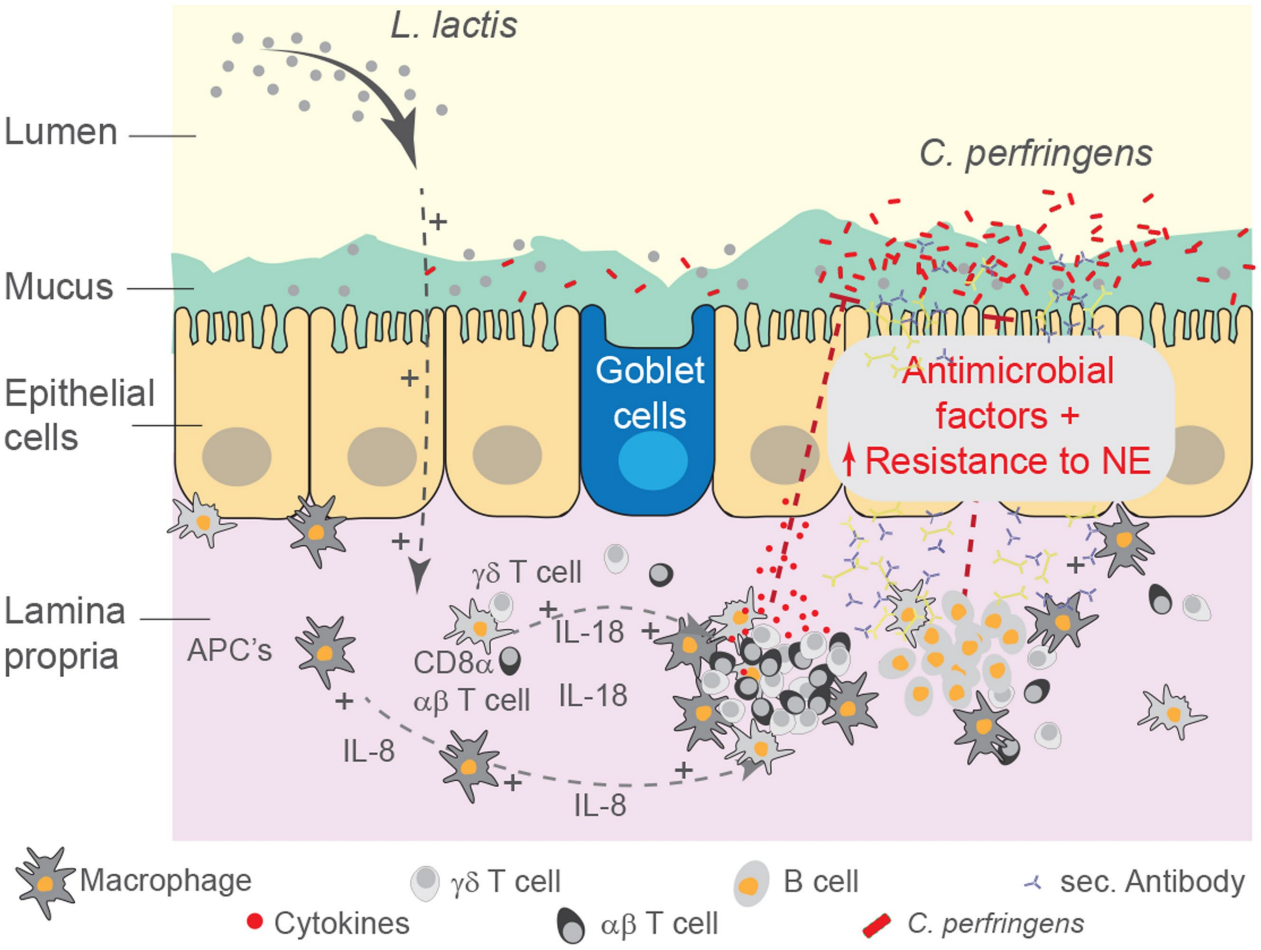
Schematic summary for the effect of *L. lactis* treatment on the intestinal mucosa. Schematic representation of the study demonstrating systematic modulation of the intestinal mucosa by *rL. lactis*. Alteration in luminal microbial content as well as intestinal immune system cells indirectly limited the severity of necrotic foci formation as a result of pathogenic *C. perfringens* infection. We speculate that *rL. lactis* promotes recruitment of monocyte-macrophages, expansion of CD8α + γδ and αβ T cells as well as IgA+ and IgY+ B cells in the lamina propria of the duodenum, jejunum and ileum. Immune cell activation, as demonstrated by changes in cytokines and chemokines, support the intestinal epithelial barrier to insult from various cytolytic toxins secreted by the pathogenic *C. perfringens*.

The intestine contains the largest number of immune system cells of any tissue, and it is continually exposed to a wide range of antigens and immune stimuli (Lee and Mazmanian, 2010). The involvement of specific host defenses, processes that take place in the mucosa and underlying lamina propria (LP), differ between microorganisms. Among several factors, pathogenic bacteria and their secretory products contribute to gut barrier impairment and its increased permeability, precisely the key predisposing conditions that facilitates *C. perfringens* cellular proliferation and progression to NE in chickens (Shojadoost et al., 2012). To counteract the effects of enteric pathogens, beneficial microbes may be employed (Alizadeh et al., 2021). Here, we evaluated the utility of *L. lactis* as a beneficial microbe whereby treatment led to a reduction in intestinal necrotic lesion scores associated with infection by a virulent *C. perfringens* strain. This observation might be due to a reduction in inflammatory processes, alteration in the proportion of intestinal immune system cell, and modification of intestinal microbial communities (Figure 11).

Indeed, the intestinal microbiota acts as a primary barrier to prevent direct contact between invading pathogens such as *C. perfringens* and the intestinal epithelium. Sequencing efforts have demonstrated that Bacillota, Bacteroidota, Pseudomonadota, Lactobacillaceae and Actinomycetota are the most abundant phyla in the human intestine with similar relative proportions found in the chicken ceca (Tap et al., 2009; Oakley et al., 2014; Clavijo and Flórez, 2018). Firstly, while *L. lactis* does not colonize the chicken intestine, treatment increased the abundance of Gram-positive (Bacillota, Lactobacillaceae and Actinomycetota) bacteria with limited changes in Gram-negative (Bacteroidota and Pseudomonadota) bacteria, in contrast to *C. perfringens* infected chickens. The latter was also observed in a mouse model whereby *C. difficile* infection increased the proportion of *Proteobacteria* and decreased Bacillota populations which correlated with more severe disease (Reeves et al., 2011). Secondly, dysbiosis can modulate TLR signaling, likely promoting *C. perfringens* mediated inflammatory processes, partly reversed by treatment with *L. lactis*. Mononuclear phagocytes active sampling of the gut microbiota modulates the GALT (Balic et al., 2014). This study demonstrates that mononuclear phagocytes make up more than one-third of all immune system cells that populate the chicken intestine which is decreased in *C. perfringens* infected chickens. Upon detecting endogenous danger signals through TLRs, mononuclear phagocytes in turn regulate B cells, and αβ or γδ T cells activity (Kono and Rock, 2008). TLR mediated and co-stimulatory signaling promote transcription of antimicrobial effector molecules (IL-17a and IL-22), regulatory factors (CTLA-4, CD40, IL-10 and TGF-β), as well as Th1 (IL-12p40, IL-18, IFN-γ) and Th2 (IL-4, IL-10 and IL-13) cytokines. TLR2 signaling has been suggested as a mechanism for dynamic feedback regulating inflammation and anti-microbial activity. Treatment with probiotic *Lactobacillus* in chickens also modulates TLR expression (Shojadoost et al., 2022). In an *ex vivo* experiment, competitive interaction by *L. lactis* has been suggested to be effective at negating TLR4-mediated modulatory effects of *C. perfringens* toxins and PAMPs on chicken intestinal immune system cells (Boodhoo et al., 2022b). Mouse studies support a protective role played by microbes that contain polysaccharide rich surface capsule that stimulate TLR2 signaling. The commensal microbiota *Bacteroides fragilis* drives differentiation of FOXP3+ T cells with regulatory function and ability to produce regulatory factors (Round and Mazmanian, 2010). The *L. lactis* NZ9000 strain used in this study also possesses a surface capsule rich in LTA motifs (TLR2 ligand) (Song et al., 2017). *L. lactis* treated chickens had higher expression of IL-10 and TGF-β. In line with these finding, the results suggested that *L. lactis* treatment can modulate intestinal immune response thereby limiting virulence associated factors of *C. perfringens*.

Selective induction of cytokines in combination with TLR stimulation modulates functions of T cells and antibody producing plasma B cells in the LP (Coffman et al., 1989). Based on FACS analysis, the chicken small intestine is populated by both CD8α + and CD4+ αβ T cell but has proportionally more CD8α + T cells at a ratio of 2.5:1. In humans, a similar ratio has been observed along the length of the small intestine (Mowat and Agace, 2014). However, *C. perfringens* infection resulted in a significant reduction in intestinal CD4+ αβ T cell while an increase in CD8α + αβ T cell was observed thereby shifting this ratio to 5:1, proportionally more in favor of CD8α + αβ T cells. *L. lactis* treatment was essential to overcome the depleting effects caused by *C. perfringens* infection on CD4+ αβ T cells populations. CTLA-4, an immune checkpoint expressed by regulatory T cells, was downregulated in *C. perfringens* infected chicken. In this study, the method of mononuclear cell isolation does not allow for differentiation between intraepithelial lymphocytes and LP lymphocytes. While these two cell types are well adapted to intestinal stimuli and perform their functions in protecting the delicate epithelial layer, the role of CD8α + αβ T cell in mucosal tissues against *C. perfringens* infection, an extracellular pathogen, is not understood.

The proportion of intestinal CD4+ to that of CD8α + αβ T cells could be a key factor to limit progression of NE. In mice, monocolonization with segmented filamentous bacteria (SFB), of the order *Clostridiales*, can induce the development of LP-resident CD4+ αβ T cells, regulatory T cells and T helper 17 (Th17) cells secreting various cytokines including TGF-β, IL-10, IL-13, IL-17a and IL-23 (Ivanov et al., 2009). Loss of mouse Th17 cells, which originate from CD4+ αβ T cells, has been implicated in microbial translocation into the intestinal epithelium and disease progression. Moreover, SFB treatment stimulates local expansion of LP-plasma cells, which secrete IgA antibodies (Ivanov et al., 2009). In addition, *Firmicutes*, which include the *Clostridia* order, are essential for induction and maintenance of tissue regulatory T cells (CD4 + CD25 + TGF-β+) during the early stages of development (Lathrop et al., 2011). The distribution and function of these T cell subsets in chickens are still unclear. However, NE severity is thought to be a result of excessive production of IL-1β, IL-13 and IL-17 cytokines during infection, a mechanism mediated via Th2 and Th17 cells (Lillehoj and Trout, 1996; Fasina and Lillehoj, 2019). Furthermore, no change in IFN-γ expression but a decrease in TGF-β expression has been observed in *C. perfringens* infected chickens (Fasina and Lillehoj, 2019; Zaytsoff et al., 2020). Expression of IFN-γ, IL-13 and IL-17 cytokines could be used as markers to define associations with NE lesion severity. This is because *C. perfringens* PAMPs and toxins likely have a dual role in activating and inhibiting specific mononuclear cells. *Ex vivo* stimulation with *C. perfringens* could effectively inhibit IFN-γ expression but upregulated expression of CD25 in intestinal T cell subsets (Boodhoo et al., 2022b). Our results are further supported based on observations whereby IFN-γ and TGF-β expression were both upregulated in *Lactobacillus* treated chickens before infection with *C. perfringens* (Brisbin et al., 2008; Shojadoost et al., 2022). IL-12p40 and IL-18 are inducers of IFN-γ which itself modulates αβ and γδ T cells, and mononuclear phagocyte functions. In the absence of IL-12p40, presence of IL-18 can stimulate Th2 cells to produce pro-inflammatory cytokines such as IL-13 (Hoshino et al., 1999; Nakahira and Nakanishi, 2011). This notion is also in agreement with our observation that infection with *C. perfringens* increased expression of IL-18 and IL-13 and not IL-12p40. Furthermore, immune mediators including leukotrienes, IFN-γ, IL-13 and IL-17a regulate production of mucus, a highly charged gel that acts as a physical barrier with direct toxic activity against many bacteria (Mowat and Agace, 2014). Overall, presence of antibacterial peptides (β-defensins and cathelicidins) and secretory antibodies (IgA and IgY) in the mucus layer serves to kill and promote their expulsion from the intestine (Mowat and Agace, 2014). Defects in mucus synthesis can lead to increased penetration of intestinal microbes into the epithelial surface. TLR2-deficient mice show impaired barrier function and more severe colitis when infected with *Campylobacter jejuni* (Stahl et al., 2014). Although the recovery of CD4+ T cell in *L. lactis* treated chickens likely contributed to improved resistance to *C. perfringens* infection, an association between IL-17/IL-13, IL-12p40/IL-18/IFN-γ, and TGF-β/IL-10 and stage of *C. perfringens* infection, before and during infection, requires further studies.

The results of the present study suggest that treatment with *L. lactis* can promote an intestinal environment which is more resistant to intestinal damage. Intestinal B cells expressing TLRs can directly sense commensal microbial associated molecular pattern (MAMPs) which results in antibody production. In addition to inducing differentiation of T cells into regulatory cells, stimulation by regulatory cytokines and co-stimulation supported by MHC class II-dependent presentation of antigens by mononuclear phagocytes promote IgA secretion by intestinal plasma B cells. The IgA isotype is the predominant immunoglobulin synthesized and secreted by LP-plasma B cells (Bos et al., 2001). Irrespective of IgA antibody specificity, active transcytosis of secretory antibodies into the mucus layer acts as an active immune barrier to prevent bacteria from penetrating into the LP (Kroese et al., 1996; Koskinen et al., 1998; Macpherson and Uhr, 2004). The former was observed in *Lactobacillus* treated chickens thereby demonstrating a conserved function to elicit a protective intestinal mucosal immune response (Haghighi et al., 2006). In this study, both IgA+ and IgY+ B cells were detected predominantly in the chicken duodenum, jejunum and ileum. The increase in both IgA+ and IgY+ B cells alone was not sufficient to limit NE progression during infection with *C. perfringens*. These results suggest that *L. lactis* treatment increased local IgA+ and IgY+ B cells frequency within the duodenum, jejunum and ileum similar to SFB and *L. lactis* treated mice (Norton et al., 1994; Ivanov et al., 2009) likely resulting in secretion of polyreactive antibodies prior to *C. perfringens* infection.

Taken together, the causal relationships and underlying mechanisms of *L. lactis* against *C. perfringens* needs to be further defined, as TLR sensing and antibody-mediated protection might be an important contributor to protective immunity.

## Data availability statement

The datasets presented in this study can be found in online repositories. The names of the repository/repositories and accession number(s) can be found in the article/supplementary material.

## Ethics statement

All animal experiments were approved (028–10,783 – ISOL and AUP 4328) by the Animal Care Committee of the University of Guelph and adhered to the guidelines for the use of animals. The study was conducted in accordance with the local legislation and institutional requirements.

## Author contributions

NB: Conceptualization, Formal analysis, Investigation, Methodology, Writing – original draft, Writing – review & editing, Data curation. BS: Data curation, Investigation, Validation, Visualization, Writing – review & editing, Methodology. MA: Formal analysis, Investigation, Writing – review & editing, Methodology. JA: Investigation, Methodology, Writing – review & editing. SB: Writing – review & editing. SS: Conceptualization, Data curation, Funding acquisition, Resources, Supervision, Validation, Writing – review & editing, Project administration, Visualization.

## Footnotes

^1^ https://www.mobitec.com/cms/download/Handbooks/NICE_Expression_System-Handbook.pdf
